# *Klebsiella pneumoniae* Oropharyngeal Carriage in Rural and Urban Vietnam and the Effect of Alcohol Consumption

**DOI:** 10.1371/journal.pone.0091999

**Published:** 2014-03-25

**Authors:** Trinh Tuyet Dao, Dror Liebenthal, Toan Khanh Tran, Bich Ngoc Thi Vu, Diep Ngoc Thi Nguyen, Huong Kieu Thi Tran, Chuc Kim Thi Nguyen, Huong Lan Thi Vu, Annette Fox, Peter Horby, Kinh Van Nguyen, Heiman F. L. Wertheim

**Affiliations:** 1 Oxford University Clinical Research Unit, Hanoi, Viet Nam; 2 Nuffield Department of Clinical Medicine, Centre for Tropical Medicine, University of Oxford, Oxford, United Kingdom; 3 National Hospital for Tropical Diseases, Hanoi, Viet Nam; 4 Hanoi Medical University, Hanoi, Vietnam; Amphia Ziekenhuis, Netherlands

## Abstract

**Introduction:**

Community acquired *K. pneumoniae* pneumonia is still common in Asia and is reportedly associated with alcohol use. Oropharyngeal carriage of *K. pneumoniae* could potentially play a role in the pathogenesis of *K. pneumoniae* pneumonia. However, little is known regarding *K. pneumoniae* oropharyngeal carriage rates and risk factors. This population-based cross-sectional study explores the association of a variety of demographic and socioeconomic factors, as well as alcohol consumption with oropharyngeal carriage of *K. pneumoniae* in Vietnam.

**Methods and Findings:**

1029 subjects were selected randomly from age, sex, and urban and rural strata. An additional 613 adult men from a rural environment were recruited and analyzed separately to determine the effects of alcohol consumption. Demographic, socioeconomic, and oropharyngeal carriage data was acquired for each subject. The overall carriage rate of *K. pneumoniae* was 14.1% (145/1029, 95% CI 12.0%–16.2%). By stepwise logistic regression, *K. pneumoniae* carriage was found to be independently associated with age (OR 1.03, 95% CI 1.02–1.04), smoking (OR 1.9, 95% CI 1.3–2.9), rural living location (OR 1.6, 95% CI 1.1–2.4), and level of weekly alcohol consumption (OR 1.7, 95% CI 1.04–2.8).

**Conclusion:**

Moderate to heavy weekly alcohol consumption, old age, smoking, and living in a rural location are all found to be associated with an increased risk of *K. pneumoniae* carriage in Vietnamese communities. Whether *K. pneumoniae* carriage is a risk factor for pneumonia needs to be elucidated.

## Introduction

*Klebsiella pneumoniae* is a gram-negative pathogen that is a common cause of both community and hospital acquired pneumonia, bacteraemia, and liver abscess in Asia [1], [2], [3]. Historically, *K. pneumoniae* has been identified as a cause of severe community acquired pneumonia, known as the Friedlander syndrome, and has been found to be particularly common in alcoholics and diabetics [1], [2], [4], [5]. Its pathogenesis is poorly understood. Alcoholics, the elderly, and diabetics can have an altered mucosal immunity, resulting in a change in the bacterial flora on their mucosal surfaces [5], [6], [7], [8], [9]. The finding that *K. pneumoniae* pharyngeal carriage rates are higher in elderly in China is likely an illustration of this phenomenon [10]. It is therefore possible that factors leading to altered mucosal immunity can affect *K. pneumoniae* colonization rates of the oropharynx. We hypothesize that oropharyngeal carriage precedes development of *K. pneumoniae* pneumonia, and that host factors, such as age and alcohol consumption, are significantly associated with carriage.

Although there have been previous studies on colonization of *Streptococcus pneumoniae* and *S. aureus* in relation to subsequent pulmonary infections [11], [12], [13], very little is known regarding oropharyngeal colonization of *K. pneumoniae* and other gram-negative bacilli (GNB). This study investigates pharyngeal *K. pneumoniae* carriage in urban versus rural Vietnamese communities and the effects of demographic and socioeconomic variables, including age, smoking habits, occupation, wealth index, history of past diseases, and alcohol consumption habits.

## Methods

### Study Sites

Ba Vi is a rural community near Hanoi City with a population of approximately 240,000. Longitudinal data collection has been performed at a demographic surveillance site (DSS) in Ba Vi (Field Laboratory Ba Vi – FilaBavi) since 1999 in 69 randomly selected clusters with about 11,000 households and 51,000 persons. Ba Vi has one district hospital and one health center in each of 32 communes [14].

Dong Da is an urban district in Hanoi with a population of about 352,000 and socioeconomic characteristics typical for urban Hanoi. An urban DSS was started in 2007 (DodaLab). The study population is 40,000 persons in 11,000 households in three selected communes with low, middle, and high socioeconomic levels. Dong Da has one district hospital and four commune health centers [14].

### Subjects

This is a cross-sectional study, in which oropharyngeal swabs were collected for bacterial culture from quota-sampled subjects from rural (Ba Vi) and urban (Dong Da) Hanoi, located in North Viet Nam. All eligible subjects (persons enrolled in the DSS) were stratified by gender and the following age categories: 1–5 years, 6–12 years, 13–19 years, 20–29 years, 30–59 years, and > = 60 years. Subjects within each age and gender category were randomly selected. As alcohol consumption is limited in women and children, we decided to only assess the role of alcohol in a separate cohort of men (19–71 years) from the Ba Vi community. Participants completed a questionnaire to identify variables possibly associated with *K. pneumoniae* carriage (Questionnaire S1). Demographic data such as age, sex, ethnicity, history of past health issues, smoking habits, daily medication intake, and antibiotic intake were recorded for each patient. Socioeconomic information including occupation, level of education, household size, and socioeconomic status was also recorded (Questionnaire S1).

### Sample Size

We aimed to enroll 2170 participants (female to male ratio of 1∶1) from the DoDa Lab and Fila Ba Vi DSS’s – *the main cohort*. An additional 850 males from Ba Vi were to be randomly selected from the list of DSS participants – *the alcohol cohort*. This sample size would allow us to estimate the prevalence rates of *K. pneumoniae* carriage across different age groups, sex, and socio-economic factors. To assess the impact of alcohol on carriage rates, heavy and moderate alcohol drinkers are compared to less-heavy drinkers. Previous data suggest that in rural Viet Nam, 24% of men are harmful drinkers, as determined by the AUDIT test developed by the WHO [15]. We estimated that a target population of 850 men would contain enough alcohol consumers to allow for meaningful analysis. Due to a higher than expected amount of enrolled patients electing to not participate, only 1029 participants were successfully included in the main cohort and 613 men were included in the alcohol cohort.

### Microbiological Methods

Separate swabs were collected for nose and throat, using sterile commercial Dacron swabs with bacteria transport medium (Copan, Italy). Nose swabs were collected from the anterior nares. Throat swabs were collected by firmly swabbing the entire posterior pharynx and tonsillar area and leaving in place for 5–10 seconds to absorb secretions, then withdrawing the swab. All swabs were transported on the same day to the microbiology laboratory of the National Hospital of Tropical Diseases in Hanoi. Within a day of arrival, the swabs were plated on MacConkey agar (Oxoid, USA). Based on morphology and Gram staining, suspected *K. pneumoniae* strains and other enterobacteriaceae were identified using API20E biochemistry strips (BioMerieux, France). All morphological unique colonies were identified and tested for antibiotic susceptibility using standard microbiological techniques. No selective media were used to enhance detection of certain resistance phenotypes.

### Statistical Analysis

*K. pneumoniae* carriage was defined as carrying *K. pneumoniae* either in nose or throat. Regarding analysis of the impact of education level: Some of the subjects had been educated according to Viet Nam’s old 10-year school system. In this case, their level of education was scaled to fit the current 12-year school system. Subjects with secondary vocational education were considered to have 10 years of education, subjects with college education were considered to have 14 years of education and those with university education were considered as having 16 years of education. If only literacy information was provided, illiterate subjects were considered as having one year of education, and literate subjects were considered as having two years of education. Wealth index is calculated by principle component analysis using of a set of correlated variables describing housing conditions (type of house, area, sanitation, water source, etc.) and household ownership assets (car, motobike, telephone, etc.).

Univariate analysis was done using Pearson’s chi-squared test or univariate logistic regression to determine p-values. Age and level of alcohol consumption were treated as continuous variables. All other variables were treated as categorical variables. For variables with a low number of positive cases (<5), Fisher’s exact test was used instead of the chi-squared test. Potential determinants of *K. pneumoniae* carriage were first considered in a univariate analysis. Variables reaching a univariate significance level of p<0.1 were included in multivariate analysis. Multivariate analysis was done through backward-stepwise regression using a binomial model with a logit link function. P-values <0.05 in multivariable analysis were considered to be significant, and odds ratios and confidence intervals for those variables were calculated. The alcohol cohort (extra 613 men from Ba Vi) was analyzed separately from the main cohort. The estimated population carriage rate is calculated by direct age-adjusted standardization using the population distribution of Viet Nam in 2011 (CIA World Factbook). All analysis was done using RStudio (RStudio, Boston, Massachusetts, USA [16].

### Ethics Statement

This study was approved by the ethical committees of Oxford University (Oxtrec), U.K. and Hanoi Medical University, Vietnam. Before participation, written informed consent from subjects or, in case of minors, their caregivers, was obtained on a standard study consent form. The consent process was approved by both ethical committees.

## Results

The number of subjects recruited for the main cohort was 1029 (53.8% male, median age: 36 years, IQR:13–53 years), with an additional 613 men from Ba Vi (median age: 47 years, IQR: 39–52) recruited for the alcohol cohort. The subjects in the main cohort were recruited somewhat equally from the original age, sex, and site strata (Table 1). Subjects that were selected but did not agree to participate were 52% male with a median age of 27 years. Young males tended to have lower participation rates, introducing a potential source of bias in the population.

**Table 1:** Distribution of study subjects, stratified by age, sex, and living location.

Age (years)	Female	Male	Dong Da	Ba Vi	Total	% of Selected
Total	467	549	394	622	1016*	46.8%
< = 5	45 (52.9%)	40 (47.1%)	26 (30.6%)	59 (69.4%)	85	3.9%
6–12	74 (44.8%)	91 (55.2%)	66 (40.0%)	99 (60.0%)	165	7.6%
13–19	60 (57.7%)	44 (42.3%)	58 (55.8%)	46 (44.2%)	104	4.8%
20–29	37 (58.7%)	26 (41.3%)	29 (46.0%)	34 (54.0%)	63	2.9%
30–59	170 (38.2%)	275 (61.8%)	126 (28.3%)	319 (71.5%)	445	20.5%
> = 60	81 (52.6%)	73 (47.4%)	89 (57.8%)	65 (42.2%)	154	7.1%

*Of the 1029 subjects recruited in the main cohort, 13 did not provide their age and are not displayed in this table. Subjects are from Dong Da (urban) and Ba Vi (rural), locations in the Ha Noi province of Northern Viet Nam. The percentages in the last column sum to 46.8% because 53.2% of the 2170 subjects selected for the study were not successfully recruited.

### Main Cohort

The overall carriage proportion of *K. pneumoniae* (either nose, throat, or both) was 14.1% (145/1029, 95% CI 12.0%–16.2%). The age-adjusted estimated carriage rate for the population of Viet Nam is 13.5% (95% CI 11.5%–15.5%). Of the positive *K. pneumoniae* cases, 15.9% (23/145) were nose carriers, 80.7% (117/145) were throat carriers, and 3.4% were carriers in both the nose and throat. *K. pneumoniae* was the most common GNB (gram-negative bacteria) isolated, accounting for 32.7% (145/443) of all GNB isolated. It was more than twice as common as any other GNB strain. Other GNB isolated included *Enterobacter cloacae* (6.1% carriage), *Enterobacter aerogenes* (2.4% carriage), and *Pseudomonas stutzeri* (1.9% carriage). Of the 145 incidences of *K. pneumoniae* identified, 6 (4.1%) were ESBL positive and 2 (1.4%) were carbapenem resistant and confirmed to be NDM1 positive. 10 cases (6.9%) were found to be cotrimoxazole-resistant.

Univariate analysis of the main cohort suggested that age (p<0.001), years of education (p<0.05), living location (urban or rural) (p<0.05), smoking (p<0.001), sex (p<0.01), history of renal diseases (p<0.01), diabetes (p<0.05), working as a farmer (p<0.05), working as a hired labourer (p<0.05), and wealth index (p<0.10) were possible candidates for being independently associated with *K. pneumoniae* carriage (Table 2). These variables were then included in backwards-stepwise linear regression until a minimal set of significant factors was achieved. Antibiotic use was also considered to determine its effects as a confounding variable. This was treated as a binary variable, and patients were distinguished based on whether they had reported taking antibiotics in the past four weeks. Antibiotic use was not found to be meaningful as a confounding variable. In the main cohort, 27.2% (280/1029) of subjects reported taking antibiotics in the past four weeks. Years of education was independently significant in subjects over the age of 18 (OR 0.93, 95% CI 0.87–0.99), but was found to be a confounding variable associated with age, as is explained below. The minimal set of significant factors was age (OR 1.03, 95% CI 1.02–1.04), smoking (OR 1.9, 95% CI 1.3–2.9), and living location (OR 1.6, 95% CI 1.1–2.4).

**Table 2:** Univariable and multivariable odds ratios for *K. pneumoniae* carriage of several variables.

	Univariable p-value	Univariable OR (95% CI)	Multivariable p-value	Multivariable OR (95% CI)
Age*	<0.001	1.03 (1.02–1.04)	<0.001	1.03 (1.02–1.04)
Years of Education*	<0.05	0.91 (0.86–0.96)	<0.05	0.93 (0.87–0.99)
Living in Ba Vi (rural)	<0.05	1.5 (1.1–2.2)	<0.05	1.6 (1.1–2.4)
Smoking	<0.001	2.7 (1.8–4.1)	<0.01	1.9 (1.3–2.9)
Male	<0.01	1.8 (1.3–2.6)	0.2	NS
Past Renal Failure	<0.01	2.4 (1.3–4.1)	0.54	NS
Diabetes	<0.05	3.1 (1.2–7.8)	0.17	NS
Farmer	<0.05	1.6 (1.0–2.4)	0.97	NS
Hired Labourer	<0.05	2.9 (1.3–6.4)	0.22	NS
Wealth Index*	<0.10	0.8 (0.6–1.0)	0.378	NS
APW*	<0.05	1.9 (1.1–3.3)	<0.05	1.7 (1.04–2.8)

*Age, years of education, wealth index, and APW (alcohol consumption per week) are analyzed as continuous variables. All other variables are categorical.

Multivariable OR’s were not calculated for non-significant (NS) variables.

For variables found to be non-significant in backwards stepwise logistic regression, the p-value reported in the 3^rd^ column is the p-value calculated when those variables were removed from the regression.

**Figure 1:**
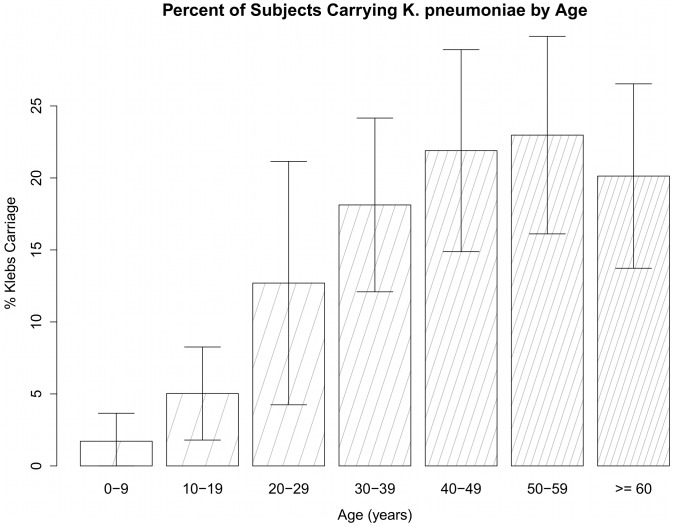
Prevalence of nasopharyngeal carriage of *K. pneumoniae* by age group – main cohort. Subjects are from Dong Da (urban) and Ba Vi (rural), two locations in Northern Viet Nam near Ha Noi. Age was found to be a significantly positively associated with *K. pneumoniae* carriage. Although age categories are used for this figure, age was analyzed as a continuous variable.

A one-year increase in age was found to be correlated with a 3% increase in odds of *K. pneumoniae* carriage (Figure 1). Carriage rates grew fairly steadily with age, from 1.8% carriage in subjects less than 10 years old to 32.5% carriage in subjects aged 50–54, but dropped off in subjects over age 54 (Figure 1).

In the main cohort, there were 174 smokers, 847 non-smokers, and 8 people that did not report smoking habits. Smokers were defined as subjects answering “yes” to the question “Do you smoke?”. The carriage rate in smokers was 26.4% (95% CI 19.8%–33.1%), compared to just 11.6% (95% CI 9.4%–13.7%) in non-smokers (p<0.01). Multivariable analysis indicated that smoking was independently associated with a 90% increase in odds of *K. pneumonia* carriage.

The participants included 399 (38.8%) subjects living in Dong Da and 630 (61.2%) subjects living in Ba Vi. The carriage rate in subjects living in Ba Vi (rural location) was 16.0% (CI 13.2%–18.9%), compared to 11.0% (CI 7.9%–14.1%) carriage in Dong Da (urban location) (p<0.05). Multivariate analysis showed that living in Ba Vi was correlated with a 60% increase in odds of carriage.

The number of years of education for each subject was only considered in subjects over the age of 18 to avoid introducing an age-dependent ceiling on education level. An increase in level of education of one year seemed to be associated with a decrease of 7% in odds of *K. pneumoniae* carriage. However, when examining the effect of education in age strata of subjects over 50 years of age and subjects between the age of 18 and 50, this effect was no longer present. Additionally, the AIC value for the model without level of education was close enough to the one with (367.06 vs. 366.23 for ages 18–50, 299.08 vs. 297.61 for ages >50) to give reasonable evidence for level of education as a confounding variable.

There were a number of variables that were positive with very low frequency (< = 5%), making their significance difficult to assess. These were diabetes (2.0%), history of heart disease (5.0%), malignancy (1.1%), HIV (0%), and past splenectomy (0.2%). Alcohol consumption information was only collected for subjects in the alcohol cohort, so we cannot assess the extent by which alcohol consumption may have confounded the results found in the main cohort. The cross-referenced frequencies of a number of variables are displayed in Table S1.

### The Effect of Alcohol Use

**Figure 2:**
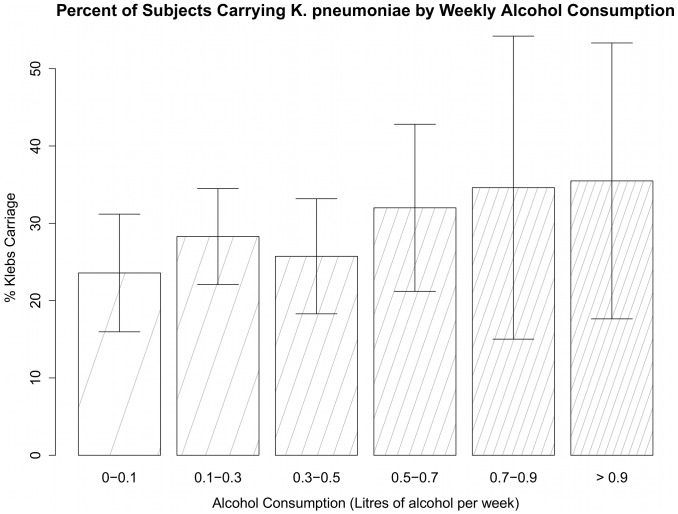
Prevalence of nasopharyngeal carriage of *K. pneumoniae* by level of weekly alcohol consumption – alcohol cohort. Subjects are from the alcohol cohort, consisting of 613 male adults from Ba Vi (rural) that provided alcohol consumption information. Alcohol consumption per week incorporates type of alcohol consumed, amount consumed, and days per week consumed.

The overall carriage rate of *K. pneumoniae* in the 613 adult men recruited to assess the effect of alcohol consumption was 28.1% (172/613, 95% CI 24.5%–31.6%). Information gathered from subjects in the alcohol cohort included type of alcohol consumed, percent alcohol content, quantity consumed per day, and days per week consumed. These categories were used to calculate the number of litres of pure alcohol consumed per week for each subject (APW), meaning that the alcohol content of the drink each subject consumed was taken into account. The median APW was 0.25 litres (range 0.0028–6.72 litres). Light drinkers were determined as subjects with APW < = 0.2, moderate drinkers had 0.2< APW < = 0.5, and heavy drinkers had APW >0.5. Using this metric, there were 235 (38.3%) light drinkers, 230 (37.5%) moderate drinkers, and 132 (21.5%) heavy drinkers. 16 (2.6%) subjects did not provide enough alcohol consumption data to calculate weekly consumption. The carriage rate was 23.0% (95% CI 17.6%–28.4%) in light drinkers, 29.6% (95% CI 23.6%–35.5%) in moderate drinkers, and 34.1% (95% CI 25.9%–42.3%) in heavy drinkers. Rate of *K. pneumoniae* carriage increased fairly steadily as level of weekly alcohol consumption increases (Figure 2).

Univariate regression analysis suggested that age (p<0.05), alcohol consumption per week (p<0.05), smoking (p<0.10), and wealth index (p<0.10) were possible significant indicators for carriage in the alcohol cohort. Alcohol consumption per week was analyzed as a continuous variable. Multivariable analysis confirmed that age (OR 1.02, 95% CI 1.003–1.05) and alcohol consumption per week (OR 1.7, 95% CI 1.04–2.8) were independently associated with carriage in this cohort. Consuming one more litre of alcohol per week was independently correlated with an increase of 70% in odds of *K. pneumoniae* carriage (Figure 2). There were just 10 (1.6%) subjects that consumed beer on a weekly basis but not hard alcohol, so the effect of the types of drinks consumed was difficult to assess. The alcohol cohort allowed us to reject the null hypothesis that *K. pneumoniae* carriage rates are equal regardless of level of alcohol consumption (p<0.05). Frequencies of *K. pneumoniae* carriage cross-tabulated with alcohol consumption information are reported in Table S1.

## Discussion

This is one of a few studies that examines oropharyngeal carriage of *K. pneumoniae* in community members, particularly in lower-middle income countries and Southeast Asia. The correlation between alcohol consumption and *K. pneumoniae* carriage has also received little prior attention. The *K. pneumoniae* oropharyngeal carriage rate of 14.1% is comparable to the nasopharyngeal carriage rate of 11% determined by a recent study in Indonesia [17]. The rate of carriage of 1.6% (95% CI 0–5.1%) in children is also comparable to a study that reported a carriage rate of 1.4% amongst children aged less than 5 years attending daycare in Brazil, an upper-middle income country (The World Bank 2013) [18].

In the past, *K. pneumoniae* carriage has been strongly linked to hospital environment-related factors such as length of hospital stay, number of artificial interventions, and duration of interventions [19]. It is commonly transmitted as a hospital-acquired pathogen, often displaying high antibacterial resistance [20]. This study aimed to identify factors associated with community based nasopharyngeal carriage of *K. pneumoniae* pneumoniae. Antibiotic use was not related to carriage which we cannot explain. Antibiotic use in the community was high, but comparable to previous studies that assessed community antibiotic use in Vietnam [21], [22], [23]. Interestingly, we found two cases who carried carbapenem resistant *K. pneumoniae* strains, which were found to be NDM-1 positive. One case was from the rural area and the other urban. We were unable to determine the source of these resistant bacteria. The ESBL-rate appears low and maybe due to the fact no selective media were used in this study.

Multiple regression analysis indicated a predicted increase in odds ratio of 3% associated with an increase in age of one year. Decreasing mucosal immunity with aging may be one mechanism underlying the association between *K. pneumoniae* carriage and aging. Similarly, a study conducted in China found that gram-negative bacilli carriage rates were relatively high in the elderly [10]. Carriage rates generally peaked in subjects aged 50–54 (28.0% carriage, 95% CI 17.6%–38.4%) (Figure 1). However, carriage rates in subjects over the age of 65 are found to be 23.9% (95% CI: 15.9%–31.9%), a decline from the rate of carriage in 50–54 year old subjects. Nevertheless, this carriage rate in the elderly matches that of the oropharyngeal carriage study done in China, which found 158 strains of *K. pneumoniae* in 706 subjects >65 years old (22.4% carriage) [10]. The reason for the observed decrease in *K. pneumoniae* carriage rates in the elderly is unknown, although it is possible that a much greater proportion of elderly carriers develop pneumonia and die, leading to an apparent decrease in carriage.

The assessment of alcohol consumption in adult men indicated that weekly alcohol consumption was positively associated with *K. pneumoniae* carriage (OR 1.7, 95% CI 1.04–2.8) (Figure 2). A prior study has found that, in Taiwan and South Africa, community-acquired bacteremic *K. pneumoniae* pneumonia is significantly associated with alcoholism [2]. Although alcoholism has been correlated with the incidence of pneumonia caused by *Klebsiella*, prior to now, very little was known about the effects of alcohol consumption on oropharyngeal colonization by *K. pneumoniae*. A likely mechanism for contracting pneumonia is aspiration of *K. pneumoniae* from the pharyngeal mucosa into the lung. Thus, the positive relationship between carriage rates and weekly alcohol consumption suggests that a potential mechanism for the increased susceptibility of alcoholics to pneumonia is the effect that alcohol has on altering the composition of oropharyngeal mucosa. This causal analysis is beyond the scope of the present study, but is worth exploring further. A prior study by Shellito et al. has identified Interleukin-17 as an important cytokine in immune response to bacterial pneumonia. The study demonstrates that ethanol consumption in mice suppresses IL-17 release into lung tissue. It suggests that IL-17 is an important part of the immunosuppression associated with alcohol abuse [5]. Another study by Zisman et al. has identified IL-12 as another cytokine that is suppressed in the lungs of mice by alcohol consumption. They have also noticed a decreased survival rate in mice with *K. pneumoniae* that were given alcohol regularly [9].

The study also found that being a smoker and living in Ba Vi (rural) were associated with an increase in risk of carriage. A recent study in Indonesia identified smoke exposure from cigarettes and irritants such as mosquito coils as being significantly associated with *K. pneumoniae* carriage. The study also found poor food and poor water hygiene as factors significantly associated with *K. pneumoniae* carriage [17]. It is reasonable that the increase in carriage rate associated with living in Ba Vi is attributable to inferior sanitation conditions. The study did not gather hygiene information, although this is a possible direction for future research. These results may be somewhat confounded by alcohol consumption. However, as alcohol consumption data was not collected for subjects in the main cohort, it is unclear whether this is the case.

These findings are a first step in elucidating potential pathways leading to community acquired pneumonia with *K. pneumoniae*. Prospective cohort studies are needed to test the hypothesis whether *K. pnaeumoniae* carriage is a risk for developing pneumonia with *K. pneumoniae*.

The results allowed us to reject the null hypothesis that risk of *Klebsiella pneumoniae* oropharyngeal carriage was independent of a variety of demographic and socioeconomic factors. Age (p<0.001), living location (p<0.05), smoking (p<0.01), and weekly alcohol consumption (p<0.05) were all found to be significantly correlated with rate of *K. pneumoniae* carriage.

## Conclusion

Oropharyngeal colonization by *K. pneumoniae* is common among healthy people. Moderate to heavy weekly alcohol consumption, old age, smoking, and living in a rural location are all found to be associated with an increased risk of *K. pneumoniae* carriage in Vietnamese communities. Whether *K. pneumoniae* carriage is a risk factor for pneumonia needs to be elucidated.

## Supporting Information

Table S1 **Cross tabulated frequencies of several variables, including** ***K. pneumoniae*** **carriage.** *K. pneumoniae* carriage frequencies are displayed, categorized by age group, smoking habits, living location, level of education, and level of alcohol consumption. Demographic information including sex, smoking habits, and living location frequencies are also displayed. Subjects are from Dong Da (urban) and Ba Vi (rural), locations in Northern Viet Nam near Ha Noi. Age, smoking, living location, level of education, and level of alcohol consumption were all found to be significantly correlated with carriage. Subjects in the “Education” rows are all over the age of 18 to avoid introducing an age-dependent ceiling on level of education. Subjects in the “Alcohol Consumption” rows are from the separate cohort of 613 adult men from Ba Vi.
(TIF)

Questionnaire S1 **Original questionnaire used to acquire socioeconomic and demographic information from study subjects.** This questionnaire was orally given to all subjects from Ba Vi and Dong Da that were recruited for the study. A trained interviewer posed the questions and recorded the responses. The questionnaire was designed to obtain information about a wide range of socioeconomic and demographic variables, allowing for more accurate assessment of associations between variables and risk of *K. pneumoniae* carriage.
(PDF)

## References

[1] Kim JK, Chung DR, Wie SH, Yoo JH, Park SW (2009) Risk factor analysis of invasive liver abscess caused by the K1 serotype Klebsiella pneumoniae. European journal of clinical microbiology & infectious diseases: official publication of the European Society of Clinical Microbiology 28: 109–111. 10.1007/s10096-008-0595-2 18663497

[2] Ko WC, Paterson DL, Sagnimeni AJ, Hansen DS, Von Gottberg A, et al (2002) Community-acquired Klebsiella pneumoniae bacteremia: global differences in clinical patterns. Emerging infectious diseases 8: 160–166. 11897067 10.3201/eid0802.010025 PMC2732457

[3] Yu VL, Hansen DS, Ko WC, Sagnimeni A, Klugman KP, et al (2007) Virulence characteristics of Klebsiella and clinical manifestations of K. pneumoniae bloodstream infections. Emerging infectious diseases 13: 986–993. 18214169 10.3201/eid1307.070187 PMC2878244

[4] Jong GM, Hsiue TR, Chen CR, Chang HY, Chen CW (1995) Rapidly fatal outcome of bacteremic Klebsiella pneumoniae pneumonia in alcoholics. Chest 107: 214–217. 7813281 10.1378/chest.107.1.214

[5] Shellito JE, quan Zheng M, Ye P, Ruan S, Shean MK, et al (2001) Effect of alcohol consumption on host release of interleukin-17 during pulmonary infection with Klebsiella pneumoniae. Alcoholism, clinical and experimental research 25: 872–881. 11410724

[6] Greenberg SS, Ouyang J, Zhao X, Parrish C, Nelson S, et al (1999) Effects of ethanol on neutrophil recruitment and lung host defense in nitric oxide synthase I and nitric oxide synthase II knockout mice. Alcoholism, clinical and experimental research 23: 1435–1445. 10512307

[7] Lau HY, Huffnagle GB, Moore TA (2008) Host and microbiota factors that control Klebsiella pneumoniae mucosal colonization in mice. Microbes and infection/Institut Pasteur 10: 1283–1290. 10.1016/j.micinf.2008.07.040 PMC2633640 18762269

[8] Quinton LJ, Nelson S, Zhang P, Happel KI, Gamble L, et al (2005) Effects of systemic and local CXC chemokine administration on the ethanol-induced suppression of pulmonary neutrophil recruitment. Alcoholism, clinical and experimental research 29: 1198–1205. 10.1097/01.alc.0000171927.66130.aa 16046875

[9] Zisman DA, Strieter RM, Kunkel SL, Tsai WC, Wilkowski JM, et al (1998) Ethanol feeding impairs innate immunity and alters the expression of Th1- and Th2-phenotype cytokines in murine Klebsiella pneumonia. Alcoholism, clinical and experimental research 22: 621–627. 10.1111/j.1530-0277.1998.tb04303.x 9622442

[10] Wang S, Li D, Chu YZ, Zhu LY, Liu FZ (2009) Determination of oropharyngeal pathogenic colonization in the elderly community. Chinese medical journal 122: 315–318. 19236811

[11] Bogaert D, De Groot R, Hermans PW (2004) Streptococcus pneumoniae colonisation: the key to pneumococcal disease. The Lancet infectious diseases 4: 144–154. 14998500 10.1016/S1473-3099(04)00938-7

[12] Brueggemann AB, Griffiths DT, Meats E, Peto T, Crook DW, et al (2003) Clonal relationships between invasive and carriage Streptococcus pneumoniae and serotype- and clone-specific differences in invasive disease potential. The Journal of infectious diseases 187: 1424–1432. 12717624 10.1086/374624

[13] Nakamura MM, McAdam AJ, Sandora TJ, Moreira KR, Lee GM (2010) Higher prevalence of pharyngeal than nasal Staphylococcus aureus carriage in pediatric intensive care units. Journal of clinical microbiology 48: 2957–2959. 20573867 10.1128/JCM.00547-10 PMC2916627

[14] Tran TK, Eriksson B, Nguyen CT, Horby P, Bondjers G, et al (2012) DodaLab: an urban health and demographic surveillance site, the first three years in Hanoi, Vietnam. Scandinavian journal of public health 40: 765–772. 23117211 10.1177/1403494812464444

[15] Giang KB, Van Minh H, Allebeck P (2013) Alcohol consumption and household expenditure on alcohol in a rural district in Vietnam. Global health action 6: 18937. 23364099 10.3402/gha.v6i0.18937 PMC3557352

[16] (2013) R: A language and environment for statistical computing. Vienna: R Foundation for Statistical Computing.

[17] Farida H, Severin JA, Gasem MH, Keuter M, van den Broek P, et al (2013) Nasopharyngeal carriage of Klebsiella pneumoniae and other Gram-negative bacilli in pneumonia-prone age groups in Semarang, Indonesia. Journal of clinical microbiology 51: 1614–1616. 23486716 10.1128/JCM.00589-13 PMC3647929

[18] Lima AB, de Oliveira Leao LS, Oliveira LS, Pimenta FC (2010) Nasopharyngeal Gram-Negative bacilli colonization in brazilian children attending day-care centers. Brazilian journal of microbiology : [publication of the Brazilian Society for Microbiology] 41: 24–27. 10.1590/S1517-83822010000100005 PMC3768626 24031458

[19] Parm U, Metsvaht T, Sepp E, Ilmoja ML, Pisarev H, et al (2011) Risk factors associated with gut and nasopharyngeal colonization by common Gram-negative species and yeasts in neonatal intensive care units patients. Early human development 87: 391–399. 21419584 10.1016/j.earlhumdev.2011.02.007

[20] Marra AR, Wey SB, Castelo A, Gales AC, Cal RG, et al (2006) Nosocomial bloodstream infections caused by Klebsiella pneumoniae: impact of extended-spectrum beta-lactamase (ESBL) production on clinical outcome in a hospital with high ESBL prevalence. BMC infectious diseases 6: 24. 16478537 10.1186/1471-2334-6-24 PMC1382232

[21] Hoa NQ, Ohman A, Lundborg CS, Chuc NT (2007) Drug use and health-seeking behavior for childhood illness in Vietnam–a qualitative study. Health policy 82: 320–329. 17118482 10.1016/j.healthpol.2006.10.005

[22] Hoan le T, Chuc NT, Ottosson E, Allebeck P (2009) Drug use among children under 5 with respiratory illness and/or diarrhoea in u rural district of Vietnam. Pharmacoepidemiology and drug safety 18: 448–453. 19326362 10.1002/pds.1730

[23] Nguyen KV, Thi Do NT, Chandna A, Nguyen TV, Pham CV, et al (2013) Antibiotic use and resistance in emerging economies: a situation analysis for Viet Nam. BMC public health 13: 1158. 24325208 10.1186/1471-2458-13-1158 PMC4116647

