# Supplementary material for: Klebsiella pneumoniae Oropharyngeal Carriage in Rural and Urban Vietnam and the Effect of Alcohol Consumption
Source: PLoS One. 2014 Mar 25;9(3):e91999. doi: 10.1371/journal.pone.0091999 (PMC3965401; doi:10.1371/journal.pone.0091999)
Supplement: Questionnaire S1 — Original questionnaire used to acquire socioeconomic and demographic information from study subjects. This questionnaire was orally given to all subjects from Ba Vi and Dong Da that were recruited for the study. A trained interviewer posed the questions and recorded the responses. The questionnaire was designed to obtain information about a wide range of socioeconomic and demographic variables, allowing for more accurate assessment of associations between variables and risk of K. pneumoniae carriage. (PDF) [file pone.0091999.s002.pdf]

Participant ID Number ☐☐☐☐☐☐☐☐

General Questionnaire:

**1. Participant ID**	☐☐☐☐☐☐☐☐		
1.1. Date of Birth or Age	_ _ / _ _ / _ _ Age ____ years<br>dd / mm/ yy		
1.2. Site	**DoDa** ☐	**Ba Vi** ☐	
1.3. Interview Date	_ _ / _ _ / _ _ (Day/Month/Year)		
1.4. Name of interviewer			
**2. Medical History**	**Yes**	**No**	**Unknown**
2.1 Diabetes	☐	☐	☐
2.2. Renal disease*	☐	☐	☐
2.3. Liver disease*	☐	☐	☐
2.4. Lung disease*	☐	☐	☐
2.5. Heart disease*	☐	☐	☐
2.6. Malignancy*	☐	☐	☐
2.7. HIV	☐	☐	☐
2.8. Tonsillectomy	☐	☐	☐
2.9. Splenectomy	☐	☐	☐
2.10 Do you smoke?	☐	☐	☐
2.11. Any other ongoing illness requiring treatment	☐ go to 2.12	☐ go to 2.13	☐ go to 2.13
2.12. Name/description of any ongoing illnesses listed			
2.13. Are you currently taking any medications?	☐ yes go to 2.14	☐ no go to 2.15	☐ don't know go to 2.15

2.14.	Name(s)	Tick if daily<br>☐<br>☐<br>☐<br>☐

2.15. Have you taken antibiotics in the past 4 weeks (See annex 3 for a list of commonly used antibiotics)?	☐yes<br>go to 2.16	☐no<br>go to 3	☐don't know<br>go to 3
2.16 If yes, specify name(s) of antibiotic:			
**3. Hanoi travel (Ba Vi site only)**	**Yes**	**No**	**Unknown**
3.1. Did you travel to central Ha Noi in the last year?	☐ go to 3.2	☐ go to 4	☐ go to 4

3.2 If yes (tick one),<br>Most days a week?<br>At least once/week but not most days?<br>At least once/month but not each week?<br>Less than monthly, but not never?<br>Don't know<br>Won't say	 ☐<br>☐<br>☐<br>☐<br>☐<br>☐

4. JE vaccination in the past	☐yes	☐no	☐don't know
5. Dengue infection in the past	☐yes	☐no	☐don't know
6. Influenza vaccination in last year	☐yes	☐no	☐don't know

Alcohol Use test

**1. Participant ID**	☐☐☐☐☐☐☐☐	
1.1 Interview Date	**DoDa** ☐	**BaVi** ☐
1.2 Name of interviewer		
Date of Interview	_ _ / _ _ / _ _ (Day/Month/Year)	

2. Do you drink alcohol once per week or more?

☐ Yes (continue) ☐ No (stop here)

If Yes, how long have you been drinking for __ __ years

If Yes, record the usual amount drunk:

Type of drink	Alcohol (%)*	Amount drunk per day (ml)†	No. days/week

*If they are not sure about % alcohol refer to the typical drink strengths chart below.

† If they are not sure about the volume drunk per day refer to typical drink sizes overleaf

Example:
If the patient drinks 4 x 330ml cans of beer (5%) every day, and 2 x 200ml glasses of whiskey (40%) on Saturday and Sunday then enter:

Type of drink	Alcohol (%)	Amount drunk per day (ml)	No. days/week
Beer	5	1320 (i.e. 330 x 4)	7
Whiskey	40	400 (i.e. 200 x 2)	2

Typical drink strengths

Beer	5%	Rice alcohol (strong)	40%
Grape wine	12%	Whiskey/spirits	40%
Rice alcohol (moderate)	30%		

Typical drink sizes

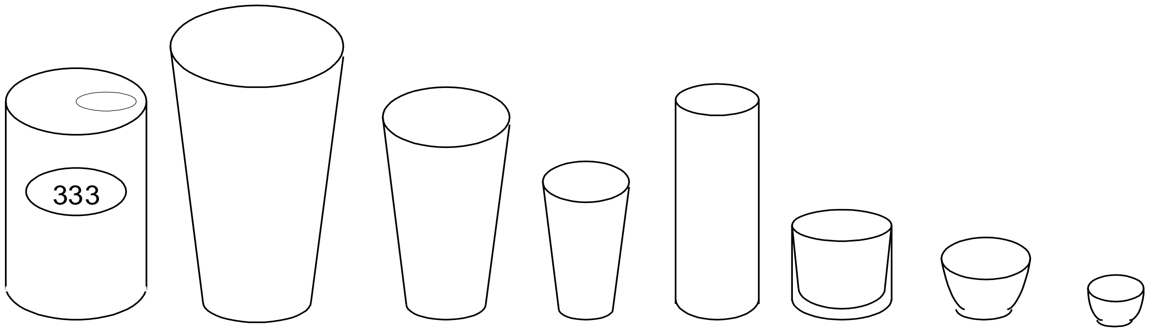

Bia lon	Cốc vại	Cốc trung	Cốc chè xanh	Cốc Kodak	Cốc Lipton	Chén nước chè	Chen hạt mít
330 ml	500 ml	350 ml	200 ml	350 ml	150 ml	80 ml	50 ml
